# Predictive value of dipstick urinalysis for detecting pyelonephritis in children aged 0–12 months

**DOI:** 10.3389/fped.2026.1777307

**Published:** 2026-03-31

**Authors:** Philippe Juvet, Jean-Yves Pauchard, Francois Cachat, Hassib Chehade

**Affiliations:** 1 Department of Pediatrics, Hôpital Riviera-Chablais, Rennaz, Switzerland; 2 Pediatric Nephrology, Centre Hospitalier Universitaire Vaudois, Lausanne, Switzerland

**Keywords:** bedside test, nephrology, primary care, pyelonephritis, urinary tract infection (UTI)

## Abstract

**Background and objectives:**

Urinary tract infections (UTIs) are the most common serious bacterial infections during the first year of life. Symptoms of UTI in young children are non-specific, making diagnosis challenging.

**Method:**

We conducted a retrospective single-center study over a period of 5 years. Urine samples collected by suprapubic aspiration, clean catch, or bladder catheterization in children aged 0–12 months presenting to the emergency department with fever without focus and suspected UTI during this period were reviewed from the laboratory archives. We divided our population into two groups of 0–3 and 4–12 months. Data on dipstick urinalysis were collected, with urine culture as the reference standard. Statistical analysis—including sensitivity, specificity, diagnostic odds ratio, likelihood ratio, positive predictive value (PPV), and negative predictive value—was performed for the following dipstick urinalysis parameters: leucocyte esterase alone, nitrites alone, leucocyte esterase and nitrites, leucocyte esterase and/or nitrites.

**Results:**

Statistical analysis showed that in the 0–3-month group, specificity was 94% for leucocyte esterase (LE) and 99% for nitrites (Nit). Sensitivity was 60% for LE and 25% for nitrites. PPV was 87% for LE and 96% for nitrites. In the 4–12-month group, specificity was 91% for LE and 98% for nitrites. Sensitivity was 71% for LE and 25% for nitrites. PPV was 82% for LE and 87% for nitrites. Combined analysis of leucocyte esterase and/or nitrites and leucocyte esterase and nitrites showed no improvement in performance.

**Conclusion:**

Dipstick analysis is a reliable bedside test for ruling in UTI in children under 12 months, particularly in the presence of positive nitrites for children less than 3 months of age. Urine culture remains necessary for diagnostic confirmation.

**Article summary:**

This study adds to the diagnostic performance of dipstick urinalysis in infants and neonates, with a specificity of 92%–99% and positive predictive value of 82%–96%.

## Introduction

Fever in children is a common presentation in both primary care and emergency departments (EDs). It can represent a diagnostic challenge, particularly in infants and neonates presenting fever without focus (1, 2). The etiology of such fever ranges from benign viral infections to serious bacterial infections, such as meningitis, occult bacteremia, and urinary tract infections (UTI) (1, 3, 4).

Urinary tract infections have their highest incidence during the first year of life (5), with reported values ranging between 5.7% and 20% (6–8). The clinical manifestations, especially in infants and neonates, are non-specific (5, 9–11).

The major challenge for clinicians is to correctly identify infants with a probable UTI and initiate prompt, appropriate treatment. Therefore, a urine sample is required for such diagnosis. While urine culture remains the gold standard for diagnosing UTI, the results are not readily available. Thus, reliable rapid testing is needed in primary care or ED settings (11). Two rapid tests are commonly available: microscopy and dipstick urinalysis. Microscopy requires trained staff, while dipstick urinalysis is easier to perform (12).

Although urine dipstick is a quick and reliable bedside screening tool in pediatrics (13–15), its diagnostic performance in young infants and neonates is not clearly established in the literature, with varying recommendations from different specialists. The British guidelines published in 2022 recommended the use of dipstick in children aged >3 months (16), whereas the American Academy of Pediatrics guidelines excluded infants aged <2 months from dipstick use recommendation in 2011 (8). However, the revised 2021 guidelines, published by Mattoo et al., did not specifically exclude this group (10).

Guidelines of the European Association of Urology/European Society of Paediatric Urology in 2014 did not specify an age threshold for dipstick use (17). The latest Swiss recommendations from 2020 suggested the use of dipstick in children aged <3 months but advised caution during result analysis due to the high voiding frequency, possibly leading to false negatives (18). Recommendations of the Pediatric Infectious Diseases Group of the French Pediatrics Society and the French-Language Infectious Diseases Society in 2015 suggested the use of dipstick starting from 3 months of age, but acknowledged studies demonstrating good diagnostic performance in children less than 3 months of age (19).

## Objectives

The objective of this study was to determine the diagnostic performance of urine dipstick analysis in children younger than 12 months. We calculated sensitivity, specificity, positive predictive value (PPV), negative predictive value (NPV), and diagnostic odds ratio (DOR) of dipstick testing in neonates and young infants (0–3 months) compared with older infants (4–12 months).

## Method

We conducted a single-center, retrospective study using data from the Children's Hospital in Lausanne (Lausanne University Hospital), Switzerland, between January 2013 and December 2018. The study was approved by the local ethics committee (BASEC 2019-00292).

We first reviewed each urine culture performed during this period from the hospital's laboratory archives and identified those obtained from children aged up to 12 months. We then reviewed the medical charts to select infants who had presented to the ED with fever without focus (fever of any duration without a clear source after complete examination) or suspected UTI.

### Definitions

Dipstick urinalysis was considered our index test, and urine culture served as our reference standard.

Based on Swiss recommendations (18), bacterial growth on urine culture was considered significant if a single germ reached ≥10^5^ colony forming unit (CFU) in clean-catch samples or ≥10^4^ CFU in urinary catheterization samples. Any single germ growth in suprapubic aspiration samples was considered significant. We classified samples as contaminated if the growth was significant but with multiple bacterial populations. We considered Leucocyte Esterase to be positive in the presence of traces to 3+. Our definitions of positive dipstick urinalysis were as follows: leucocyte esterase alone, nitrites alone, leucocyte esterase and nitrites, leucocyte esterase, and/or nitrites.

### Inclusion criteria

We included children aged 0–12 months who underwent both dipstick urinalysis (index test) and urine culture (reference standard) in the context of a consultation in the ED for fever without focus or suspected UTI. Urine specimens had to be obtained through suprapubic aspiration, clean catch, or bladder catheterization.

### Exclusion criteria

We excluded children whose urinalysis was performed in another context (e.g., voiding cystourethrography, post-operative) and those who did not meet the criteria of fever without focus or suspected UTI (i.e., absence of fever, other diagnosis, or no reported diagnosis in the medical charts).

Children whose urine sampling has been collected by bag or for whom urine culture or dipstick analysis data were missing were also excluded.

### Group division

In addition to dipstick analysis and urine culture, we collected clinical data (age, gender, collection method) and divided the children into two groups. The first group involved children aged 0–3 months and the second involved children aged 4–12 months. The division into these two subgroups was decided based on the fact that children aged <3months have the highest prevalence of serious bacterial infections overall (from 10% up to 15%–22%), as reported in the literature (6, 20).

### Statistical analysis

We performed statistical analysis (sensitivity, specificity, PPVs, NPVs, and DOR) for both groups for the following definitions of positive dipstick urinalysis: leucocyte esterase alone, nitrites alone, leucocyte esterase and nitrites, and leucocyte esterase and/or nitrites.

Likelihood ratios were also calculated. Ninety-five percent CIs were estimated using Wald's or Wilson's method, as appropriate.

## Results

**Figure 1:**
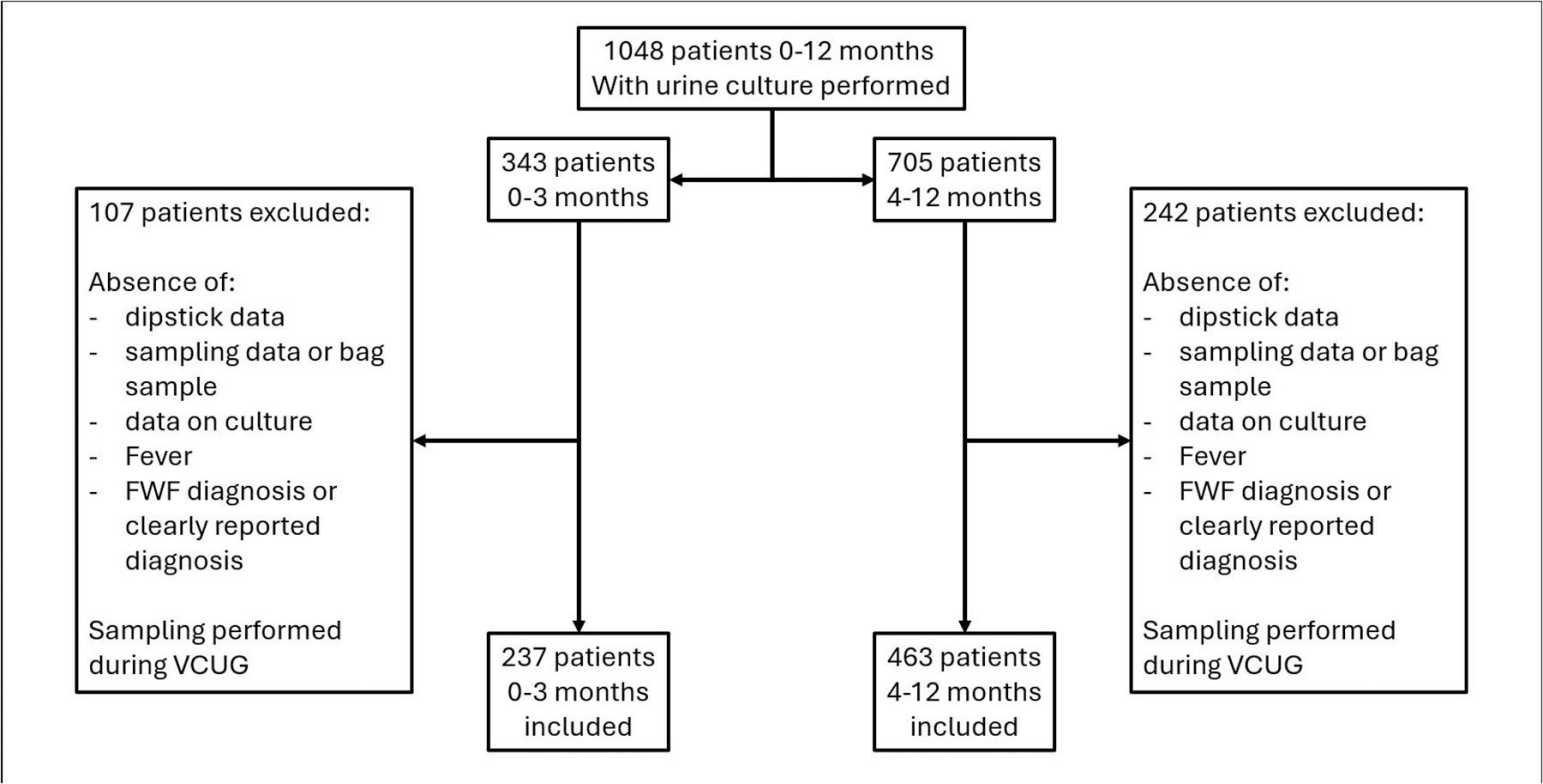
Flowchart showing the inclusion and exclusion criteria for patients. FWF, fever without focus; VCUG, voiding cystoureterography.

We analyzed data on 1,048 samples collected between January 2013 and December 2018. Of these, 349 samples were excluded, with the reasons for exclusion provided in the flowchart in Figure 1. In both groups, contaminated cultures were classified as negative.

Population characteristics are summarized in Table 1. Ethnicity was not documented in the clinical records.

**Table 1:** Description of population characteristics.

	UTI		No UTI	
	*N*	%	*N*	%
0–3 months				
Total	96		140	
Sex				
Male	72	*75* *.* *00*	68	48.57
Female	24	*25* *.* *00*	72	51.43
Sampling				
CC	16	*16* *.* *67*	10	7.14
BC	80	*83* *.* *33*	130	92.86
4–12 months				
Total	162		301	
Sex				
Male	85	*52* *.* *47*	138	45.85
Female	77	*47*.*53*	163	54.15
Sampling				
CC	30	*18*.*52*	30	9.97
BC	132	*81*.*48*	271	90.03

UTI, urinary tract infection; CC, clean catch; BC, bladder catheterization. Percentages represent column percentages.

### Group 1

0–3 months (*N* = 236): UTI 96 (40.68%): This group was composed of 59.3% boys. Urine collection was realized by following methods: bladder catheterization: 210 (89%); clean catch: 26 (11%).

A total of 96 urine cultures showed significant bacterial growth, and 14 samples were contaminated. The results of the statistical analysis of dipstick urinalysis are presented in Table 2.

**Table 2:** Statistical analysis of dipstick urinalysis for subgroups 0–3 and 4–12 months.

	Sensitivity	95% CI	Specificity	95% CI	PPV	95% CI	NPV	95% CI	DOR	95% CI	LR +	95% CI	LR −	95% CI
0–3 months														
* *LE	0,60	0.51–0.70	0.94	0.9–0.98	0.87	0.78–0.95	0.78	0.71–0.84	22.22	10.09–48.93	9.4	4.27–20.70	0.42	0.19–0.93
* *Nit	0.25	0.16–0.34	0.99	0.96–1.0	0.96	0.80–0.99	0.66	0.59–0.72	46.33	6.14–349.47	35.0	4.64–263.99	0.76	0.1–5.7
* *LE and Nit	0.25	0.16–0.34	0.99	0.96–1.0	0.96	0.80–0.99	0.66	0.59–0.72	46.33	6.14–349.47	35.0	4.64–263.99	0.76	0.1–5.7
* *LE and/or Nit	0.60	0.51–0.70	0.94	0.9–0.98	0.87	0.78–0.95	0.78	0.71–0.84	22.22	10.09–48.93	9.4	4.27–20.70	0.42	0.19–0.93
4–12 months														
* *LE	0.71	0.64–0.78	0.91	0.88–0.95	0.82	0.75–0.88	0.85	0.82–0.89	25.88	15.29–43.80	8.22	4.86–13.91	0.32	0.19–0.54
* *Nit	0.25	0.18–0.31	0.98	0.96–1.0	0.87	0.77–0.97	0.71	0.66–0.75	16.12	6.66–39.01	12.39	5.12–29.97	0.77	0.32–1.86
* *LE and Nit	0.23	0.16–0.29	0.98	0.97–1.0	0.88	0.78–0.98	0.7	0.66–0.75	17.52	6.73–45.63	13.75	5.28–35.80	0.78	0.30–2.04
* *LE and/or Nit	0.73	*0.66* *–* *0.80*	0.91	*0.88* *–* *0.94*	0.81	*0.75* *–* *0.88*	0.86	*0.82* *–* *0.90*	27.22	*16.09* *–* *46.03*	*8* *.* *12*	*4.8* *–* *13.73*	0.3	*0.18* *–* *0.5*

LE, leucocyte esterase; Nit, nitrites.

Positive leucocyte esterase had a sensitivity of 0.6 (95% CI 0.51–0.70), specificity of 0.94 (95% CI 0.9–0.98), PPV of 0.87 (95% CI 0.78–0.95), NPV of 0.78 (95% CI 0.71–0.84), DOR of 22.22 (95% CI 10.09–48.93), likelihood ratio (LR) + of 9.4 (95% CI 4.27–20.70), and LR– of 0.42 (95% CI 0.19–0.93).

Positive nitrites had a sensitivity of 0.25 (95% CI 0.16–0.034), specificity of 0.99 (95% CI 0.96–1.0), PPV of 0.96 (95% CI 0.80–0.99), NPV of 0.66 (95% CI 0.59–0.72), DOR of 46.33 (95% CI 6.14–349.47), LR + of 35 (95% CI 4.64–263.99), and LR− of 0.76 (95% CI 0.10–5.70).

Combined analysis of leucocyte esterase and nitrites, i.e., “Leucocyte esterase and/or nitrite positive” and “Leucocyte esterase and nitrite positive,” showed the same results. Indeed, all patients with positive nitrites also had positive leucocyte esterase.

### Group 2

4–12 months (*N* = 481): UTI 163 (33.9%): This group was composed of 48.16% boys. Urine collection was realized by the following methods: catheterization: 403 (87.04%); clean catch: 60 (12.96%). A total of 162 urine cultures showed significant bacterial growth, and 24 were contaminated.

The results of the statistical analysis of dipstick urinalysis are provided in Table 2.

Positive leucocyte esterase had a sensitivity of 0.71 (95% CI 0.64–0.78), specificity of 0.91 (95% CI 0.88–0.95), PPV of 0.82 (95% CI 0.75–0.88), NPV of 0.85 (95% CI 0.82–0.89), DOR of 25.88 (95% CI 15.29–43.80), LR + 8.22 (95% CI 4.86–13.91), and LR– 0.32 (95% CI 0.19–0.54).

Positive nitrite had a sensitivity of 0.25 (95% CI 0.18–0.31), specificity of 0.98 (95% CI 0.96–1.0), PPV of 0.87 (95% CI 0.11–0.97), NPV of 0.71 (95% CI 0.66–0.75), DOR of 16.12 (95% CI 6.66–39.01), LR + of 12.39 (95% CI 5.12–29.97), and LR− of 0.77 (95% CI 0.32–1.86).

The combined analysis of leucocyte esterase and nitrites, i.e., “Leucocyte esterase and/or nitrite positive” and “Leucocyte esterase and nitrite positive,” showed no improvement in sensitivity, specificity, NPV, PPV, or DOR.

## Discussion

The sensitivity (25% in both groups) and specificity (99% in the 0–3-month group and 98% in the 4–12 month-group) values for nitrites in our study were similar to the ones reported by Suresh et al. (21), Marques et al. (22), and Lendner et al. (23). In contrast, our sensitivity values for leucocyte esterase were lower than expected [60% in our study vs. 79% (1), 88% (24), and 92% (21) in other studies with a similar age group]. High specificity for both leucocyte esterase and nitrites was comparable across the two groups (0–3 and 4–12 months). Tzimenatos et al. (25) reported higher sensitivity (92%) and specificity (96%) for leucocyte esterase, and a specificity of 99% for nitrites, in children with UTI in a retrospective multicenter study of 26 ED centers and a population of infants aged <60 days.

A meta-analysis by Coulthard on 18 studies suggested that nitrite sticks are not safe to use for screening in children aged <2 years, as they may miss up to 75% of UTI. The same analysis also suggested that a positive nitrite result is very likely to indicate UTI at any age. The role of leucocyte esterase was not evaluated in this meta-analysis (26).

Our findings align with the results of earlier studies (26, 27) suggesting that nitrites seem particularly useful for ruling in acute pyelonephritis in children <4 months if positive, with a positive predictive value of 96%. In the 4–12-month group, the positive predictive rate for nitrites was 87%. In children <4 months presenting with suspected acute pyelonephritis or fever without focus and positive nitrites, only 4% would not have required antibiotics, compared with 13% in children aged 4–12 months.

However, our data do not suggest the use of negative nitrites on dipstick urinalysis to rule out urinary tract infections in either group, given the false-negative rates of 34% and 29% in the 0–3- and 4–12-month groups, respectively. This could be explained by the fact that nitrites are less readily found in the urine of small children due to frequent bladder voiding. Nitrates require a few hours to be reduced to nitrites in the presence of uropathogens carrying nitrate reductase enzymes (11, 21, 28).

Lendner et al. published a case-controlled study highlighting high false-negative rates in dipstick testing, defining a positive dipstick as positive leucocyte esterase and/or nitrites (23). Similar results emerged from Reardon et al.'s study, performed in 2008, on well-appearing febrile children <2 years, showing poor results for urinalysis with a sensitivity of 64% and a specificity of 91%. Their results did not provide details for each part of the urinalysis [pyuria on microscopy, leucocyte esterase (LE) and nitrites on dipstick] (27).

On the other hand, some studies have reported promising results for dipstick urinalysis in young infants. A study performed by Glissmeyer et al. (29) focused specifically on children aged <90 days and supported the use of dipstick analysis (combined for LE and nitrites) with a good sensitivity of 90.8 (95% CI 90.4–91.2) and specificity of 93.8% (95% CI 93.5–94.1). A study by Waterfield et al. (30), focusing solely on infants aged <3 months, reported varying sensitivity and specificity depending on the definition of a positive dipstick for leucocyte esterase and nitrites, but supported its use in this age group.

In our study, combined analysis (leucocyte and nitrites and leucocyte AND/OR nitrites) showed no gain in sensitivity or specificity in the 0–3-month group, since all samples with positive nitrites also had positive leucocyte esterase. In the 4–12-month group, there was a small gain in sensitivity and specificity on combined analysis. The positive predictive value for leucocyte esterase and nitrites was high in our 0–3-month group (87% and 96%, respectively). The DOR and positive likelihood ratio were also high but with a broad 95% CI.

Our data support dipstick urinalysis as a good tool to rule in acute pyelonephritis in children between 0 and 12 months, especially if nitrites are positive in children aged <4 months. However, the use of urine culture remains necessary to confirm the diagnosis, identify uropathogens, and adapt the antibiotic regimen to the bacteria's antibiotic sensitivity. Importantly, the data of this study do not support the use of dipstick urinalysis to rule out UTI.

The strength of our study lies in its large sample size of young infants, which enabled us to confirm that dipstick urine analysis is an effective and quick bedside tool in primary analysis and diagnostic decisions. To our knowledge, few studies have analyzed in detail the diagnostic performance of individual dipstick urinalysis parameters exclusively in a cohort of young children. The limitations of the present study include its retrospective and monocentric design and missing information, which restricted exploration of all set objectives and reduced the available study population.

## Conclusion

Dipstick analysis is a reliable bedside test for ruling in UTI, particularly if positive for nitrites in infants <4 months. However, urine culture remains necessary for diagnostic confirmation, as it is impossible to draw conclusions on the pathogen by solely relying on dipstick analysis.

Future prospective studies should aim to combine clinical signs and symptoms with urine and blood sampling analyses to develop a diagnostic algorithm for UTI in neonates and infants.

## Data Availability

The datasets presented in this study can be found in online repositories. The names of the repository/repositories and accession number(s) are as follows: https://doi.org/10.5281/zenodo.17607760.
